# The functional property of royal jelly 10-hydroxy-2-decenoic acid as a melanogenesis inhibitor

**DOI:** 10.1186/s12906-017-1888-8

**Published:** 2017-08-09

**Authors:** Chi-Chung Peng, Hui-Tzu Sun, I-Ping Lin, Ping-Chung Kuo, Jen-Chieh Li

**Affiliations:** 1 Department of Biotechnology, National Formosa University, Huwei, Yunlin Taiwan; 2 Department of Research and Development, Challenge Bioproducts Co., Ltd., Douliou, Yunlin Taiwan; 3 Honey Bee Town Co., Ltd., No.77-2 Huaxi, 970 Hualien City, Taiwan

**Keywords:** Royal jelly, 10-hydroxy-2-decenoic acid, Melanogenesis, Skin-whitening, Melanogenesis inhibitor

## Abstract

**Background:**

It has been reported that royal jelly would reduce melanin synthesis and inhibit the expression of melanogensis related proteins and genes. In this study, we evaluate the anti-melanogenic and depigmenting activity of 10-hydroxy-2-decenoic acid (10-HDA) from royal jelly of *Apis mellifera*.

**Methods:**

In this study, we assesses the 10-HDA whitening activity in comparison with the changes in the intracellular tyrosinase activity, melanin content and melanin production related protein levles in B16F1 melanoma cells after treating with 10-HDA. Furthermore, the skin whitening effect was evaluated by applying a cream product containing with 0.5%, 1% and 2% of 10-HDA onto the skin of mice (C57BL/6 J) for 3 week to observe the effect of DL*-values.

**Results:**

The results showed that 10-HDA inhibited the MITF protein expression (IC50 0.86 mM) in B16F1 melanoma cells. Western blot analysis revealed that 10-HDA inhibited the activity of tyrosinase and the expression of tyrosinase-related protein 1 (TRP-1), TRP-2, and microphthalmia-associated transcription factor (MITF) in B16F1 melanoma cells. In addition, the 10-HDA was applied on the skin of mice show significantly increased the average skin-whitening index (L value).

**Conclusions:**

The validation data indicated the potential of 10-HDA for use in suppressing skin pigmentation. The 10-HDA is proposed as a candidate to inhibit melanogenesis, thus it could be developed as cosmetics skin care products.

## Background

Royal jelly (RJ) is young worker bees (*Apis mellifera*) secretion produced by the hypopharyngeal and mandibular glands, the developing queen bee was fed it exclusively for her lifetime [1]. RJ provides high nutritional values owing to the abundant amounts of proteins, free amino acids, lipids, vitamins, and sugars [2, 3]. The bioactive proteins of RJ are the major royal jelly proteins (MRJPs), apisimin and royalisin, which have shown immunoregulatory and antibacterial effects in several studies [4–6]. 10-hrdroxy-2-decenoic acid (10-HDA) was the major fatty acid in RJ possess several health-beneficial effects for human, which has demonstrated antitumor, antibacterial, and immunomodulatory activities [7–9]. 10-HDA is only found in RJ so it has been used as a quality marker of royal jelly products [10, 11]. Several pharmacological activities of RJ have already been confirmed by animal experiments, and the pharmacological activities including anti-oxidation [12, 13], anti-inflammation [14], anti-tumor [15, 16], anti-agenesis [17], antibacterial [18–20], vasodilative [21, 22], hypertensive [21, 22], anti-hypercholesterolemic [23], nephroprotective [24] and skin-whitening effects [25]. Based on its nutritional value and its benefit for human health there are more and more commercial RJ products are available in the markets.

The skin color of animals and humans is related to the content of melanin pigment in the skin. The role of melanin is to protect skin against the damage from UV-light, but excessive accumulation of melanin causes serious skin disorders such as discoloration, pigmented and accelerated skin aging [26]. Melanin was synthesizeds in the melanocytes located in the innermost layer of the epidermis via melanogenesis mechanisms [27]. Melanogenesis is a complex biosynthetic pathway controlled by tyrosinase, tyrosinase-related protein 1 and 2 (TRP-1 and TRP-2) and microphthalmia-associated transcription factor (MITF) [28, 29]. Tyrosinase is a rate-limiting enzyme for controlling the synthesis of melanin. The first step of melanin production is the hydroxylation of L-tyrosine to L-3,4-dihydroxyphenylalanine (L-DOPA) and the conversion of L-DOPA to dopaquinone [30]. TRP-2 catalyzes the production of 5,6-dihydroxyindole-carboxylic acid converted from dopachrome, and the product of TRP-2, 5,6- dihydroxyindole-carboxylic acid as a substrate for TRP-1 converted to indole-5,6-quinone carboxylic acid, ultimately resulting in melanin synthesis [31, 32]. To inhibit melanin synthesis via disruption melanogenesis would be the major way to prevent or improve hyperpigmentary disorders, such as melasma, and age spots. Therefore, searching for a potential, safe and effective compound to down-regulate in those factors in melanogenesis would be noteworthy in medical and cosmetic industry [25, 33, 34].

Royal jelly has been demonstrated that it could reduce melanin synthesis [22], but the major active compound or the mechanism underlying these activities of RJ remains unknown. In our previous study, we found that 10-HDA could inhibit the tyrosinase activity (unpublished data). In this study, the inhibitory effect on typrosinase by 10-HDA was further evaluated. Melanin biosynthesis in vitro models using B16F10 melanoma cell culture and an animal model of mouse with the skin application were both performed to investigate the melanogenesis-inhibiting effect of 10-HDA.

## Methods

### Preparation of 10-HDA from royal jelly

Royal jelly was prepared by the Fu-Chang Beekeeping in Hualien, Taiwan. Larvae of 3-day ages were transferred into queen cell cups on the frames, and each frame contained 30 queen cups. The frames were transferred into bee hives, and the RJ was collected 72 h after transferring the larvae. Each hive contains approximately 25,000 of honeybees [35]. The collected RJ samples were kept at −20 °C until further analysis. Royal jelly (40 g) was refluxed with methanol (400 mL × 4 × 30 min), and the supernatant was harvested via centrifugation at 4500 x g for 30 min. The supernatant was concentrated under a reduced pressure to obtain the crude extract (10.76 g) named RJM. RJM suspended in methanol was purified by silica gel column chromatography (SiO2 CC), and then eluted with chloroform and methanol gradients (300:1 to 1:1) to obtain ten fractions analyzed by TLC. Fractions 4 and 5 displayed significant spots, and therefore they were subjected to the further purification and analysis. Fraction 4 and 5 were repeatedly purified by SiO2 CC (eluted with chloroform/methanol, 300:1 to 1:1) and further recrystallized with acetone to get 10-hydroxy-2-decenoic acid (10-HDA). The chemical structure of the purified product was confirmed by NMR and GC-mass spectra analysis. The 10-HDA quantitative analysis was determined by high-performance liquid chromatography (HPLC) with a Waters 1525 pumping system equipped with a Water 2489 detector, an RP-8 GP250 column (4.6 mm) and a Waters 717plus autosampler. The mobile phase was methanol solution (60:40 *v*/v with ultrapure and deionized water) adjusted with phosphoric acid to pH 2.5, filtered through a membrane (0.45 μm) and degassed for 5 min. The mobile phase flow-rate was adjusted to 1.0 mL/min, and the detection was performed at 225 nm. The content of 10HDA in the final purified sample is 90%, which is used for further analysis.

### Cell culture and 10-HDA treatments

B16F10 melanoma cells (BCRC number: 60,031) were cultured in Dulbecco’s modified Eagle’s medium (DMEM) supplemented with 10% (*v*/v) fetal bovine serum at 37 °C in a humidified, CO2-controlled (5%) incubator. The cells were seeded at an appropriate cell density in a 24-well or a 6-well plate. After 1 d of incubation, the cells were treated with various concentrations of 10-HDA. Medium was used in the control group instead of 10-HDA. Thereafter, the cells were harvested and used for various assays.

### Measurement of cell viability

Cell viability was measured by 3-(4,5-dimethylthiazol-2-yl)-2,5 -diphenyltetrazolium bromide (MTT) assay according to the method reported by Carmichael et al. [36]. B16F10 melanoma cells were cultured in DMEM containing 10% FBS and 1% L-glutamine (4 mM) in a 5% CO_2_ incubator at 37 °C. Cultured cells (1 × 10^4^ cells/well) were seeded in a 96-well plate, 10-HDA (dissolved in dimethyl sulfoxide (DMSO)) diluted by the medium at a concentration of 1, 0.5, 0.1 mM and kojic acid 1 mM were added to the wells. The medium was used as the blank. After 24-h incubation at 37 °C under 5% CO_2_, the media was removed from each well, then the wells were washed with PBS (1 M phosphate-buffer saline) for two times. 200 μl of MTT solution (2 mg/ml) was added to each well. The reaction was terminated by adding 100 μL of DMSO after 4-h incubation. The absorbance of each well was measured at 540 nm using an immunoassay reader (BIO-TEK, Winooski, VT) [20–23]. Cell viability was determined by the following equation: Cell viability (%) = [(A-B)/C] × 100%, A:sample absorbance volume, B: blank absorbance volume, C: control absorbance volume.

### Measurement of cellular melanin content

Intracellular melanin content of B16F10 melanoma cells was measured using the modified method described by Bilodeau et al., [37]. At the end of B16F10 melanoma cell culturing, the cells were harvested and washed with PBS. The harvested cells were lysed in cold lysis buffer (20 mM sodium phosphate (pH 6.8), 1% Triton X-100, 1 mM phenylmethylsulfonyl fluoride (PMSF), 1 mM Ethylenediaminetetraacetic acid (EDTA)). After centrifugation at 15,000×*g* for 30 min, the pellets were dissolved in 1 N NaOH containing 20% DMSO for 1 h at 60 °C. The protein content in the supernatant was determined using the Bradford assay. The absorbance at 405 nm was measured, and the melanin content was calculated against a known standard of synthetic melanin. Melanin level (%) = [(A-B)/C] × 100%; A: sample absorbance volume; B: blank absorbance volume; C: control absorbance volume.

### Measurement of cellular tyrosinase activity

A tyrosinase activity assay was performed according to the method described previously by Martinez-Esparza et al., [38], with slight modifications. B16F10 melanoma cells were lysed in 20 mM sodium phosphate (pH 6.8), 1% Triton X-100 and 1 mM phenylmethane sulfonyl fluoride or PMSF, and centrifuged at 14,000 rpm for 15 min. The protein content of each supernatant was determined using the Bradford assay with Bovine Serum Albumin (BSA) as the protein standard. Tyrosinase activity was determined in a reaction mixture (1 mL) containing 50 mM phosphate buffer (pH 6.8), 2 mM L-DOPA and 300 μg supernatant proteins. After incubating at 37 °C for 15 min, the absorbance at 475 nm was measured using a microplate reader. Tyrosinase activity (%) = [(A-B)/C] × 100%; A: sample absorbance volume; B: blank absorbance volume; C: control absorbance volume.

### Western blot analysis

The cells were washed 3 times in ice-cold PBS and lysed in RIPA buffer (pH 7.4, 50 mM tris, 0.1% SDS, 50 mM NaCl, 1% NP-40, 1 mM PMSF, 10 μg/mL aprotinin and 10 μg/mL leupeptin). An aliquot of the lysate was used to determine the protein content by the method of Bradford assay using bovine serum albumins as the standard. The proteins (40 μg) were separated on 10% SDS-polyacrylamide gel electrophoresis and blotted onto Hybond-C Extra nitrocellulose membrane (Amersham Bioscience, U.K.). The membranes were blocked with 5% non-fat milk in Tris-buffer saline (TBS) containing 10 mM Tris-HCl, pH 7.5 and 150 mM NaCl for 30 min. MITF, tyrosinase, dopachrome tautomerase 2 (TRP-2), TRP-1 and β-actin (as an internal control) were detected using a rabbit polyclonal antibodies, respectively. The membranes were further incubated with goat polyclonal secondary antibody to rabbit IgG-H&L (HRP). All bound antibodies were then detected using Super Signal® West Pico Chemiluminescent Substrate (ECL) (Thermo Scientific). The signal intensity of each band was quantified with a densitometer system Gel Doc TM / Chemi Doc TM Universal hood II (Bio-Rad) equipped with an integrator, and normalized with that of the internal control.

### Determination of depigmenting activity in mice

The depigmenting activity was assay via the mouse model system by the modified protocol of Tai et al. [39]. The study was approved by the Ethics Committee of National Formosa University (approval number: 10,401). Five-week-old female black mice (C57BL/6 J), weighing 20 to 25 g, were purchased from the National Animal Laboratory Center, Taipei. Throughout all experiment, animals were housed in an air-conditioned room with constant temperature (25 °C ± 2 °C) and kept on a 12 h light: dark cycle. The animals were acclimatized for 7 days prior to the experiment. After shaving their hair, the animals were given 1-day rest. Gel samples containing 10-HDA were prepared by dispersing the drugs in Vaseline. A total of forty mice were equally divided into five groups and each group was smeared twice daily with 0.1 g Vaseline (control), 1% kojic acid in Vaseline, 0.5%, 1% or 2% 10-HDA in Vaseline. The applications continued for 3 weeks, and the skin-whitening index (L value) was measured on the same skin area every day with a DermaLab® Combo (Cotex Technology, Denmark), which is a colorimetric instrument that uses a high intensity white LED as a light source. The colorimetric instrument is connected to a computer [40]. We considered only the L parameter, and the L-value was the relative brightness, ranging from total black (L = 0) to total white (L = 100). The initial skin-whitening index L-value was assayed from the skin of each mouse before being applied with the tested substances.

### Statistical analysis

All results in this study were analyzed using the general linear-model procedure available from Statistical Analysis System software package version 9.1 (Statistical Analysis System Institute, 2002). Duncan’s multiple range test [41] was used to detect differences between the means of the treatments. Each experiment was conducted in triplicates.

## Results

### Effect of 10-HDA on B16F1 melanoma cell viability

**Fig. 1:**
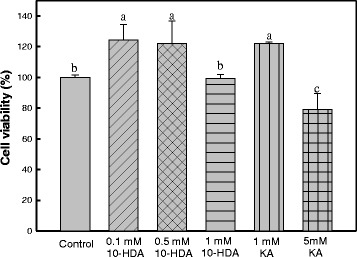
Effect of 10-HDA on B16F10 melanoma cell viability. a–c Different letters among samples indicate significant differences (*P* < 0.05)

The optimal dose from the cell viability assay by MTT in B16F1 melanoma cells are shown in Fig. 1. It clearly showed that 10-HDA was not cytotoxic to B16F1 melanoma cells at concentrations of 0.1, 0.5 and 1 mM, and kojic acid was not cytoxic to melanoma cells at a concentration of 1 mM. Cell viability decreased significantly (20% reduction) in melanoma cells exposed to 1.5 mM 10-HDA (*P* < 0.05) (data not shown) and 5 mM kojic acid. Therefore, the concentrations of 0.1, 0.5, 1 mM of 10-HDA and 1 mM of kojic acid were applied in subsequent experiments.

### Inhibition of tyrosinase activity and melanin synthesis in B16F10 melanoma cells by 10-HDA

**Fig. 2:**
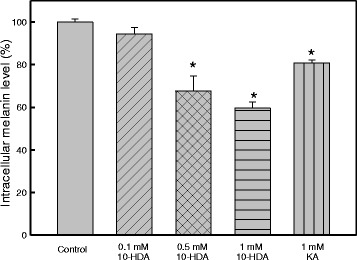
The inhibitory effect on melanogenesis in B16F1 cells. B16F1 melanoma cells were treated with different concentrations of 10-HDA. Data are presented as mean ± SEM of three independent experiments. *P < 0.05 is considered to be significant

**Fig. 3:**
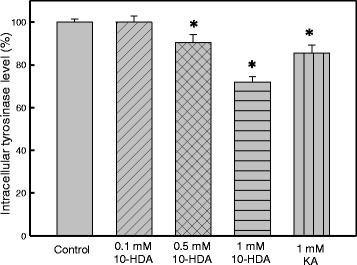
Inhibitory effect of 10-HDA on tyrosinase activity in B16F1 cells. Cellular tyrosinase activity was measured using B16F1 melanoma cell lysates. Data are presented as mean ± SEM of three independent experiments. Different symbol indicates a significant difference. *P < 0.05 is considered to be significant

Kojic acid is an effective and well-known anti-melanogenesis agent, and was used as a positive control in this study. 10-HDA significantly (*p* < 0.05) suppressed melanin synthesis and tyrosinase activity compared to the control of the non-treated B16F1 melanoma cells. At the dose of 1 mM, 10-HDA induced 28 ± 2.4% reduction in cellular tyrosinase activity (*P* < 0.01) and 40.4 ± 3.0% reduction in cellular melanin synthesis (*P* < 0.001), while kojic acid (1 mM) also significantly reduced tyrosinase activity and melanin synthesis by 14.4 ± 3.7% (*P* < 0.05) and 19.3 ± 1.5% (*P* < 0.001), respectively (Figs. 2 and 3).

### Suppression of tyrosinase, TRP-1, and TRP-2 protein expression in B16F10 melanoma cells by 10-HDA

**Fig. 4:**
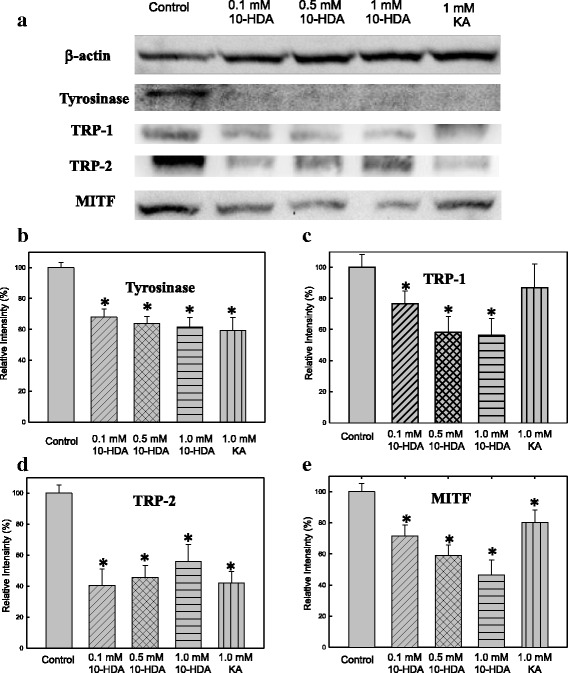
Effect of 10-HDA on melanogenic protein expression in B16F1 melanoma cells. Cellular protein expression patterns in the B16F1 melanoma cell lysates were analyzed by western blot: (**a**) Densitometric analysis was performed using NIH image analysis software for (**b**) tyrosinase, (**c**) TRP-1, (**d**) TRP-2 and (**e**) MITF. The relative band intensity of the enzymes was normalized by β-actin. * *P* < 0.05 is considered to be significant

To investigate whether 10-HDA can influence melanogenic protein expression, Western blotting analysis was carried out using the lysate of B16F10 melanoma cells treated with 10-HDA (Fig. 4). The 10-HDA dramatically inhibited tyrosinase, TRP-1 and TRP-2 expressions in B16F1 melanoma cells compared with those of the untreated cells (Fig. 4b–d). The β-actin, a housekeeping protein that was used as an internal control, showed no change. The 10-HDA inhibited the protein expression levels of melanogenic enzymes as similar to kojic acid.

### Inhibitory effect of the 10-HDA on the protein level related to melanogenic factors in the B16F10 cells

During the process of melanogenesis in mammalian cells, MITF plays the major regulator role of the synthesis of TRPs pathway, including TYR, TRP-1, and TRP-2 [25, 26]. The effect of the 10-HDA on MITF expression was evaluated using Western blotting. B16F10 melanoma cells were exposed to various concentrations of 10-HDA (0.1, 0.5 and 1 mM), resulting in the down regulation of MITF expression by 10-HAD (Fig. 4e). The IC50 value for 10-HDA suppression of MITF expression was estimated to be 0.86 mM. The present results suggest that MITF protein levels are reduced by the 10-HDA. The hypopigmentation effect of the 10-HDA may be the result of down-regulated MITF gene expression, which would then represses the protein and gene expressions of tyrosinase, TRP-1 and TRP-2.

### Evaluation of depigmenting activity of 10-HDA in vivo via mice

**Fig. 5:**
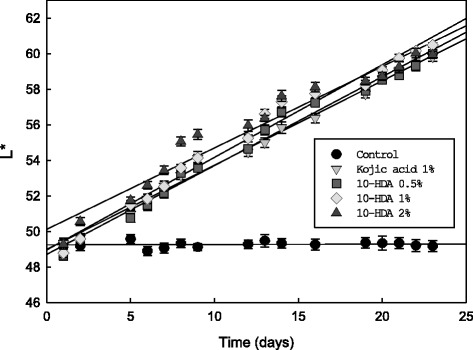
Time course of skin whitening index (L value) of mice treated with Vaseline only (control, ), 1% Kojic acid in Vaseline (), 2% 10-HDA (), 1% 10-HDA () or 0.5% 10-HDA () in Vaseline. A total of forty mice were equally divided into five groups, and each group was smeared twice daily in 5 consecutive days a week for 3 weeks with 0.1 g Vaseline (control, ), 1.5% Kojic acid in Vaseline (), 2% 10-HDA (), 1% 10-HDA () or 0.5% 10-HDA () in Vaseline. The L value was measured once a day on the same skin area with a DermaLab® Combo. Averaged L values (*n* = 8) with an error bar of SD are plotted over the experimental period

In order to speculate the human dosage, we used mice as an animal model to investigate the depigmenting activity of 10-HDA. After shaving, mice were treated with 1% kojic acid in Vaseline, 0.5%, 1% or 2% 10-HDA in Vaseline, and the skin lightening index was measured and recorded. For this in vivo study, we used kojic acid as a positive control. Kojic acid is widely used as a skin depigmentation therapy agent around the world. After the first week of treatment, the skin whitening degree was significantly increased in the mice treated with 10-HDA, compared to the control, and this increase continued until the end of the experiment. The depigmenting activity of 0.5, 1 and 2% 10-HDA was comparable to that of 1% kojic acid. Our results revealed that 10-HDA was able to significantly promote the skin whitening on mice skin at as low as a concentration of 0.5% (Fig. 5). Therefore, 10-HDA appears to be a good candidate as a skin whitening agent to treat skin hyperpigmentation.

## Discussion

Melanin synthesis is controlled by the complex enzymatic cascade of tyrosinase, TRP1 and TRP2. The degree of melanogenesis-related gene and protein inhibition play an important role in the efficacy of a depigmenting agent, which is usually utilized in the treatment of hyperpigmentation or cosmetic products [28]. To elucidate the true inhibitory effect of 10-HDA on melanogenesis, the melanin content and intracellular tyrosinase activity of the B16F10 cells were assayed at the same concentration range. The results in Figs. 2 and 3 indicated that the 10-HDA exhibited a higher inhibit activity on melanin synthesis in B16F10 cells than kojic acid. The data revealed that 10-HDA blocks melanogenesis in B16F10 melanoma cells.

Melanogenesis is dominated at least by three regulatory proteins, tyrosinase, TRP1 and TRP2 in mammalian melanocytes [29]. The expressions of TRP-1, TRP-2, and MITF were all inhibited in the B16F10 melanoma cells, which were treated with 10-HDA. MITF is a major transcription factor to regulate the expression of melanogenic enzymes, such as tyrosinase, TRP-1 and TRP-2 [42, 43]. According our Western blotting data (Fig. 4), mammalian cell treat with 10-HDA would reduce the expression of all rate-limiting enzymes, including tyrosinase, TRP-1, and TRP-2, and prevented abnormal accumulation of melanin duing the melanogenesis process. These data suggested that 10-HDA could inhibit the process of melanogenesis by inhibiting MITF expression. In this study we have demonstrate that 10-HDA inhibits melanogenesis by downregulating of MITF protein, tyrosinase and melanin production. The inhibitory pathway of 10-HDA on MITF expression was different from the other melanogenesis inhibitors, such as kojic acid, arbutin, and ascorbic acid, which had no effect on MITF expression [44, 45]. This suggest that 10-HDA has an excellent potential to be used as a safe and natural skin-whitening agent for functional cosmetics [46].

Melanoma, a skin cancer arise from the malignant transformed of the melanocytes. Melanomas arising in chronically sun-damaged skin, mucosal surfaces, and acral skin were cause by over-activate BRAF and NRAS [47], loss of the CDKN2A locus [48], overexpressed the MITF [49], overactivate Kit [50], over-activate mGluR1 [51, 52]…et al. In this study, 10-HDA could inhibit MITF expression in B16F10 melanoma cells. This indicated that 10-HDA has potential to be the ingredient for dermal medicine or anticancer against melanoma.

RJ has been used for many dermatological preparations include skin refreshing, skin regeneration, rejuvenation, burn healing or wounds healing [53, 54]. Moreover, it was reported that some unsaturated fatty acids in RJ could inhibit melanin synthesis and tyrosinase activity, leading to downregulation of melanogenesis [55], and we demonstrated that the depigmentation effect of RJ might be resulted from the presence of 10-HDA. Because the skin of mice is similar to human, thus we used mice as an in vivo animal model for examining the depigmenting activity of 10-HAD. As shown in Fig. 5, the skin whitening index of skin color was significantly increased in mice treated with 10-HAD, compared to the control. In addition, Koya-Miyata et al. were evaluate collagen production-promoting activity of 10-HDA in fibroblast cell line by produce transforming growth factor-β1 (TGF-β1), which is an important factor for collagen production [55]. Therefore, 10-HDA appears to be a very promising natural compound for skin regeneration and hyperpigmentation treatment.

## Conclusion

10-HDA inhibited not only the tyrosinase activity, but also the melanogenic enzyme expressions, including tyrosinase, TRP-1 and TRP-2, by suppressing MITF in the B16F10 melanoma cells. Consequently, the pigment of melanin was reduced in B16F10 melanoma cells. Furthermore, the in vivo animal model showed the depigmenting activity of 10-HDA in topical application. These results suggested that 10-HDA has a candidate could be a safe and natural melanogenesis inhibitor for the cosmetics industry, in which searching a natural and effective biocompound is one of the very important objective for developing a better skin care product.

## Abbreviations

10-HDA: 10-hydroxy-2-decenoic acid
BSA: Bovine Serum Albumin
DMEM: Dulbecco’s modified Eagle’s medium
DMSO: Dimethyl sulfoxide
EDTA: Ethylenediaminetetraacetic acid
L-DOPA: L-3,4-dihydroxyphenylalanine
MITF: microphthalmia-associated transcription factor
MRJP: major royal jelly proteins
MTT: 3-(4,5-dimethylthiazol-2-yl)-2,5-diphenyltetrazolium bromide
PBS: phosphate-buffer saline
PMSF: phenylmethane sulfonyl fluoride or phenylmethylsulfonyl fluoride
TLC: thin layer chromatography
TRP-1: tyrosinase-related protein 1
TRP-2: tyrosinase-related protein 2
